# Feedback from physical activity monitors is not compatible with current recommendations: A recalibration study

**DOI:** 10.1016/j.ypmed.2016.06.017

**Published:** 2016-10

**Authors:** Dylan Thompson, Alan M. Batterham, Oliver J. Peacock, Max J. Western, Rahuman Booso

**Affiliations:** a Department for Health, University of Bath, Bath BA2 7AY, UK; b Health and Social Care Institute, Teesside University, Middlesbrough TS1 3BA, UK; c Directorate of Health Services, Air Force Head Quarters, P.O. BOX 1592, Colombo 02, Sri Lanka

**Keywords:** Physical activity status, Physical activity recommendations, Physical activity monitoring, Physical activity energy expenditure, Exercise

## Abstract

Wearable devices to self-monitor physical activity have become popular with individuals and healthcare practitioners as a route to the prevention of chronic disease. It is not currently possible to reconcile feedback from these devices with activity recommendations because the guidelines refer to the amount of activity required on top of normal lifestyle activities (e.g., 150 minutes of moderate-to-vigorous intensity activity per week over-and-above normal moderate-to-vigorous lifestyle activities). The aim of this study was to recalibrate the feedback from self-monitoring.

We pooled data from four studies conducted between 2006 and 2014 in patients and volunteers from the community that included both sophisticated measures of physical activity and 10-year risk for cardiovascular disease and type 2 diabetes (n = 305). We determined the amount of moderate-to-vigorous intensity activity that corresponded to FAO/WHO/UNU guidance for a required PAL of 1.75 (Total Energy Expenditure/Basal Metabolic Rate).

Our results show that, at the UK median PAL, total moderate-to-vigorous intensity physical activity will be around 735 minutes per week (~ 11% of waking time). We estimate that a 4% increase in moderate-to-vigorous intensity activity will achieve standardised guidance from FAO/WHO/UNU and will require ~ 1000 minutes of moderate-to-vigorous intensity activity per week.

This study demonstrates that feedback from sophisticated wearable devices is incompatible with current physical activity recommendations. Without adjustment, people will erroneously form the view that they are exceeding recommendations by several fold. A more appropriate target from self-monitoring that accounts for normal moderate-to-vigorous lifestyle activities is ~ 1000 minutes per week, which represents ~ 15% of waking time.

## Highlights

• Many people are self-monitoring their physical activity in a way that was impossible in the past.
• Current physical activity guidelines were not formulated with such technology in mind.
• Our study shows that self-monitoring is not compatible with current activity guidelines.
• An adjusted target for self-monitoring is ~ 1000 min of activity per week (~ 15% of waking time).

## 1 Background

There has been an explosion in the availability of wearable devices that allow people to self-monitor and track their physical activity (Chiauzzi et al., 2015). Wearable technologies are enormously popular and it is estimated that in 2016 alone global sales will approach 100 million units (Statista, 2015). Thus, there are millions of people around the world who are beginning to self-monitor their physical activity in a way that was never possible in the past.

These wearable technologies are a potentially very useful way for individuals to self-monitor and manage their physical activity as a route to the prevention of chronic disease (Thompson et al., 2015, Western et al., 2015). In addition, as the accuracy and affordability of these technologies has improved, they are beginning to play an increasingly important role in healthcare and public health (Chiauzzi et al., 2015).

Based on our previous research (Thompson et al., 2015, Thompson and Batterham, 2013, Thompson et al., 2009), we suspected that many people will receive feedback from physical activity monitoring that is difficult to reconcile with recommended levels of physical activity from national agencies (e.g., The Department of Health in the UK, (Department of Health, 2011)). The public as well as healthcare providers need to be equipped with an understanding of the output from these devices if they are to be used successfully to help support and/or monitor behaviour.

The purpose of the present investigation was to clarify and recalibrate physical activity feedback from wearable technologies in order to reconcile differences with physical activity recommendations and thus provide guidance to help the public and healthcare practitioners interpret this potentially valuable information.

## 2 Methods

### 2.1 Design

To ensure that this research was not prone to either device- or population-specific influences, we combined data from studies that used two very different devices for the collection of physical activity data and that also targeted different populations (including both the general public and patients recruited from primary care). We collated data from several studies that were conducted at the University of Bath between 2006 and 2014. In all studies, sophisticated measures of physical activity were employed to characterise participants and other measures were also included to enable the calculation of cardiovascular and type 2 diabetes risk (QRISK and/or QDiabetes score). One analysis draws on studies that recruited men from the local community and used an expensive research-grade instrument for the assessment of physical activity energy expenditure (Comparison 1). The second analysis comes from a study that recruited patients from primary care and employed a commercially-available physical activity monitor (Comparison 2). A key feature of this analysis is that the instruments used in both comparisons derive accurate estimates of physical activity in units of energy expenditure (kJ/min). Participants in all studies provided written informed consent.

### 2.2 Comparison 1: research-based physical activity monitor

For comparison 1, we examined our previous projects to identify studies where we had both physical activity data and risk of cardiovascular disease and type 2 diabetes in middle-aged participants from the local community. We identified three studies and pooled these data to undertake the present comparison (Thompson et al., 2010, Dixon et al., 2009, Travers et al., 2014). The device employed to collect physical activity energy expenditure in these studies was a research instrument that uses synchronized accelerometry and heart rate with branched equation modelling (Actiheart, Cambridge Neurotechnology Ltd., Cambridge, UK) and has been shown to have excellent accuracy (Brage et al., 2005, Brage et al., 2004, Thompson et al., 2006, Crouter et al., 2007). Each device costs around £1000 ($1500 US) and it is unlikely that this specific instrument will ever be sold directly to the public. However, given the rate of technological progress, commercially-available wearable technologies will be likely to perform at a similar level in the near future.

In all three studies for comparison 1, participants were recruited from the local community via advertisement (National Health Service Research Ethics Committee reference numbers 06/Q2001/30, 06/Q2001/105, and 11/SW/0193). Participants were non-smoking men who were not taking any medication and were aged 35 to 64 years (Table 1). In two studies (n = 66), participants were only included if they self-reported low participation in structured exercise (i.e., two or fewer occasions of structured exercise lasting 30 minutes per week). One study recruited a sub-group of highly active volunteers who self-reported participation in at least 30 minutes of moderate intensity physical activity per day plus vigorous intensity exercise at least 3 times per week (n = 12). In the remaining volunteers (n = 23), no specific physical activity or exercise inclusion criteria were employed.

### 2.3 Comparison 2: commercially-available physical activity monitor

For comparison 2, we undertook an analysis of physical activity data from patients recruited from primary care in the South West of the UK as part of the Mi-PACT trial (National Health Service REC reference number 13/SW/0179). The men and women in this study were recruited based on having moderate-to-high risk of cardiovascular disease or type 2 diabetes according to records in GP databases. The physical activity data were collected with a physical activity monitor that is already being sold and used widely by the public (BodyMedia FIT, BodyMedia Inc., Pittsburgh, PA). Importantly, unlike many other commercially-available devices, it is possible to extract raw minute-by-minute estimates of energy expenditure in order to undertake the necessary data processing to extract the key physical activity metrics required for our analysis (SenseWear® Pro 8.0, algorithm v5.2). Although this technology is available to the public as a commercial product, it has also been used in research studies and has excellent reported accuracy (Shuger et al., 2011, Welk et al., 2007, Scheers and Philippaerts, 2013, Johannsen et al., 2010, Bond et al., 2014, Lee et al., 2014).

The sample for this comparison represents the first 204 sequential patients who were screened as part of the Mi-PACT study (Peacock et al., 2015). Briefly, this study recruited men and women from primary care aged 40–70 years at medium (≥ 10 and < 20%) or high (≥ 20%) risk of cardiovascular disease and/or type II diabetes mellitus (based on QRISK or QDiabetes scores calculated from records in their GP's notes). People were excluded with existing coronary heart disease, chronic kidney disease (stages 3–5), diabetes mellitus, stroke, heart failure and peripheral arterial disease. Participant characteristics are shown in Table 1.

### 2.4 Physical activity energy expenditure: data handling and analysis

Both physical activity instruments used in the current analysis are body mounted and collect data on a minute-by-minute basis (day and night). Weekly physical activity energy expenditure records for both comparisons were exported to Excel and processed in exactly the same way. To be included, physical activity data was required for at least 90% of a full 7-day period. Missing data were allocated estimated energy expenditure equivalent to basal metabolic rate (Schofield et al., 1985). We determined Physical Activity Level (PAL) as the product of Total Energy Expenditure/Basal Metabolic Rate (Thompson et al., 2009) and time engaged in moderate intensity activity (> 3 Metabolic Equivalents or METs) and vigorous intensity activity (> 6 METs), where one MET represents resting metabolic rate. The primary analysis focuses on total accumulated physical activity data – the supplementary section online includes an analysis using bouts of activity > 10 minutes (Additional File 3).

### 2.5 QRISK and QDiabetes

We used age, sex, ethnicity, smoking status, cholesterol/HDL ratio, systolic blood pressure and body mass index to estimate QRISK and QDiabetes scores for each participant using the combined QIntervention platform (ClinRisk, 2013).

### 2.6 Data analysis

Statistical analysis was conducted in 2015. We used simple linear regression to determine the average amount of physical activity per week above 3 METs associated with a PAL of 1.75 – the level of physical activity recommended by the FAO/WHO/UNU (FAO/WHO/UNU, 2004). This model was applied to comparisons 1 and 2, separately, and to a pooled data set of all 305 participants. For simplicity, no study or participant characteristics were included in the models; this decision made no material difference to the estimates. Also, in the pooled model alone, we determined the amount of activity that an individual person would need to accumulate such that the probability that their true PAL was ≥ 1.75 was at least 95% (“very likely”; (Hopkins et al., 2009)). Using the standard error of the estimate obtained by regressing PAL on minutes per week > 3 METS, we derived the minutes per week necessary for the lower limit of the 90% individual prediction interval for PAL to be 1.75. Applying a reference Bayesian approach, if the lower limit of the 90% confidence interval is 1.75 then the probability that this individual's true PAL is ≥ 1.75 is 95% (area to the right of 1.75 = 0.95).

## 3 Results

As shown in Table 1, participants spent the majority of the time (~ 9000 minutes per week) engaged in activities below the moderate-to-vigorous intensity threshold of 3 METs (i.e., they spent most of their time engaged in sedentary activities, light intensity activities and sleep).

### 3.1 Moderate-to-vigorous intensity physical activity (comparison 1 and 2)

Total weekly moderate-to-vigorous intensity physical activity was 852 ± 386 minutes for comparison 1 and 1090 ± 571 minutes for comparison 2 (Table 1). Thus, in spite of using different devices and different populations, the accumulated total amount of moderate-to-vigorous intensity physical activity as a proportion of the week was broadly similar across the two comparisons. It is noteworthy that every person in the present study recorded > 150 minutes of moderate-to-vigorous intensity physical activity per week. The full dataset is available in the supplementary section online (Individual-level data for Comparison 1, Individual-level data for Comparison 2).

**Fig. 1:**
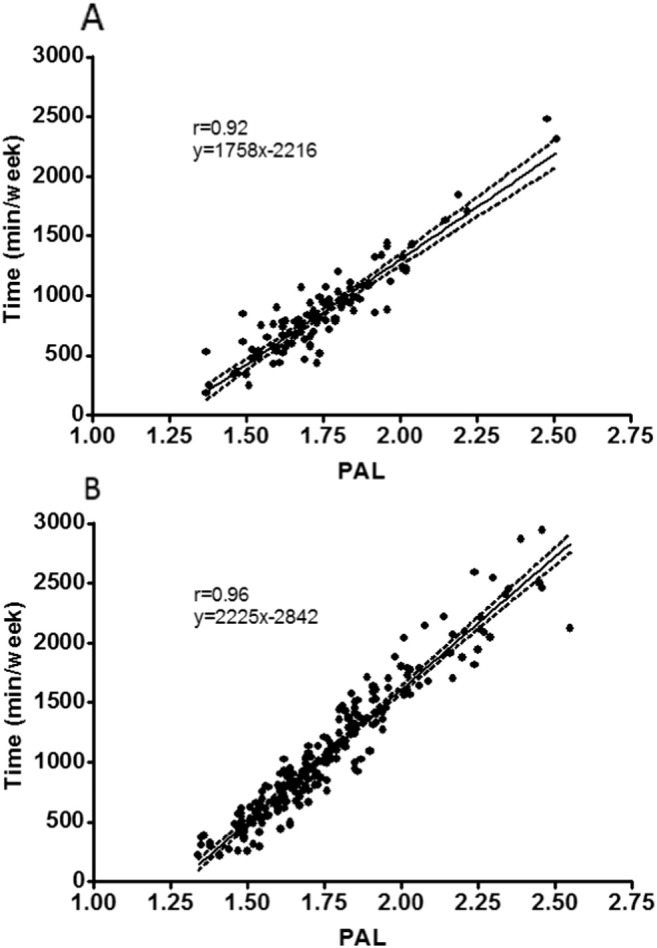
The relationship between PAL as a standardised measure of physical activity energy expenditure (TEE/BMR) and time engaged in moderate-to-vigorous intensity physical activity. Panel A shows the research instrument used in Comparison 1 (n = 101) and Panel B the commercial instrument used in Comparison 2 (n = 204). Data was collected between 2006 and 2014. The regression line with 95% CI are included. Inspection of residuals plots revealed no problems with model specification, other than one substantial outlier in comparison 2, with a negative standardised residual > 4 standard deviations from the mean. A sensitivity analysis revealed that removal of this case made no material difference to the findings, so we elected to retain it in the analyses.

The relationship between standardised physical activity energy expenditure in the form of PAL and the amount of time spent engaged in moderate-to-vigorous physical activity is shown in Fig. 1. Using these regression equations, a PAL of 1.75 as advocated by FAO/WHO/UNU equates to 861 and 1052 minutes of moderate-to-vigorous physical activity for comparisons 1 and 2, respectively.

### 3.2 Moderate-to-vigorous intensity physical activity (merged across both comparisons)

**Fig. 2:**
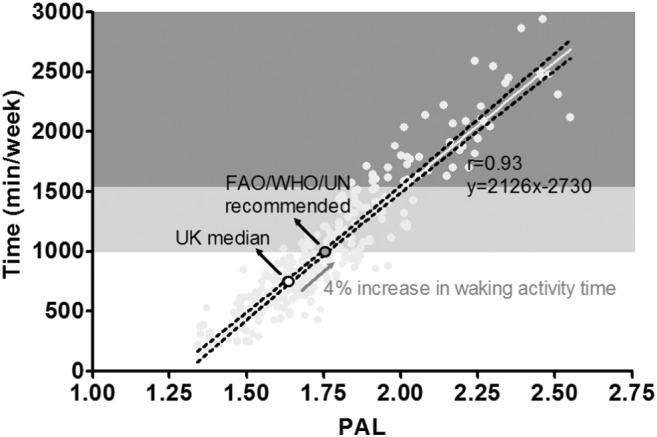
The relationship between PAL and time engaged in moderate-to-vigorous intensity physical activity merged across both comparisons (n = 305). Data was collected between 2006 and 2014. The UK median PAL is displayed (1.63) in addition to the FAO/WHO/UN recommended PAL of 1.75. The light grey and dark grey shaded areas depict ranges for ‘moderately active’ and ‘vigorously active’ lifestyles as set out by FAO/WHO/UN (FAO/WHO/UNU, 2004).

We merged the data for both comparisons to determine the relationship between standardised physical activity energy expenditure in the form of PAL and the amount of time engaged in moderate-to-vigorous intensity physical activity (Fig. 2; n = 305). A comprehensive assessment of energy expenditure in the UK calculated median PAL to be 1.63 and this is used as the basis for current UK energy requirements (Scientific Advisory Committee for Nutrition, 2011). Based on our analysis shown in Fig. 2, we estimate that an increase from the UK median PAL of 1.63 to a PAL of 1.75 as advocated by FAOWHO/UNU would require an increase in moderate-to-vigorous physical activity of 4% of waking time assuming a 16 h waking day (i.e., from 11% to 15% of total waking time). For someone at the UK median PAL, this would require an increase in moderate-to-vigorous intensity physical activity of around 255 minutes per week on top of existing physical activity.

To put this into context given our focus on self-monitoring using wearable devices, we estimate that the *total* amount of moderate-to-vigorous intensity activity required to achieve a PAL of 1.75 is 990 minutes per week based on our merged analysis across both comparisons (95% confidence interval for the predicted mean, 969 to 1012 minutes).

The standard error of the estimate in predicting PAL from minutes per week of moderate to vigorous physical activity was 0.08 units. The amount of moderate-to-vigorous intensity activity associated with a lower limit of the 90% individual prediction interval for a PAL of 1.75 was 1330 minutes per week: point estimate for PAL = 1.89 (90% individual prediction interval 1.75 to 2.03). Thus, for a 95% probability that an individual's true PAL is ≥ 1.75 requires an accumulated total of 1330 minutes of moderate-to-vigorous physical activity per week.

## 4 Discussion

Our results demonstrate that data from accurate physical activity monitoring produces a picture that is incompatible with the 150 minute per week target disseminated via national public-facing websites (NHS Choices, 2013). Instead, when using such devices, a more appropriate target is ~ 1000 minutes of moderate-to-vigorous intensity physical activity per week.

In this study, we show that average moderate-to-vigorous intensity physical activity from sophisticated self-monitoring is 5 to 7-fold greater than the 150-minute per week target advocated by key healthcare agencies (NHS Choices, 2013, The Department of Health, 2012, Canadian Society for Exercise Physiology, 2012). This finding is robust and consistent across two different populations and using two different devices. The reason for this mismatch is because the 150-minute target was originally proposed to be on top of ‘baseline’ physical activity, which is defined as ‘normal lifestyle activities’ (Powell et al., 2011, Office of Disease Prevention and Health Promotion, 2008). As previously discussed by Powell et al., this concept has been rather soft and poorly characterised in the past because of the limited evidence that was available at the time (Powell et al., 2011). Perhaps as a result of this uncertainty, the reference to ‘on top of baseline’ is usually omitted from wider dissemination aimed at the public (NHS Choices, 2013, The Department of Health, 2012, Canadian Society for Exercise Physiology, 2012). This means that the perceived target for weekly physical activity has become 150 minutes, which is reasonable until people start using sophisticated instruments that capture ALL their physical activity (i.e., including baseline normal moderate-to-vigorous lifestyle activities). Our analysis anchors the output from sophisticated technologies to thresholds from the FAO/WHO/UN (FAO/WHO/UNU, 2004, World Health Organisation, 2000), and will help avoid confusion by the public and healthcare professionals.

The median PAL in the UK is 1.63 (Scientific Advisory Committee for Nutrition, 2011) and this is not hugely different to the median PAL across both comparisons reported in the present study (1.72). Thus, these observations are not because we have somehow recruited an unrepresentative sample. In this context, it is noteworthy that recent large observational studies in hundreds of participants from across Europe and North America demonstrate that weekly moderate-to-vigorous intensity physical activity is 600–1200 minutes when assessed using sophisticated research instruments (Thompson and Batterham, 2013, Thompson et al., 2009, Scheers and Philippaerts, 2013, InterAct Consortium, 2012, Scheers et al., 2012, Drenowatz et al., 2015). It is particularly noteworthy that every person in the present study, even those with a very low PAL (e.g., < 1.39), exceeded 150 minutes of accumulated moderate-to-vigorous intensity physical activity per week. This mismatch is important because most commercially available devices report the sum of accumulated moderate-to-vigorous intensity physical activity as the primary feedback. Thus, self-monitoring with sophisticated physical activity monitors will not provide a picture that is compatible with current physical activity recommendations.

We have previously proposed that the output from technological innovation in physical activity monitoring will require recalibration if viewed in the context of physical activity recommendations (Thompson and Batterham, 2013, Thompson et al., 2009). Our current analysis goes some way towards achieving this goal. We show that, for someone at the UK median PAL, moderate-to-vigorous intensity activity will be around 735 minutes per week (equivalent to ~ 11% of waking time, assuming 8 h of sleep). This approximates typical ‘baseline’ moderate-to-vigorous intensity activity; and a 4% increase in waking moderate-to-vigorous intensity activity would be required to shift PAL to the level recommended by the FAO/WHO/UN (Fig. 2). Our derived relationship is entirely consistent with an independent analysis from the Institute of Medicine (IOM) (Brooks et al., 2004). The IOM estimated that to increase PAL from their defined sedentary level of 1.39 to their low active category of 1.60 would require an increase in daily moderate-to-vigorous intensity physical activity of 60 minutes per day. Our estimate, based on the relationship shown in Fig. 2, would require a remarkably similar increase of 64 minutes per day (i.e., to increase PAL from 1.39 to 1.60). Thus, after factoring in normal ‘baseline’ lifestyle activities, it appears that approximately 1000 minutes of moderate-to-vigorous intensity physical activity per week corresponds to standardised guidance from the FAO/WHO/UN (FAO/WHO/UNU, 2004, World Health Organisation, 2000). This may initially seem like a large amount of activity, but it only represents ~ 15% of weekly waking time (with the remaining 85% distributed across sedentary and light intensity activities). This does not mean that people need to start doing 1000 minutes of ‘new’ moderate-to-vigorous intensity activity – instead, it represents the sum of all activities after accounting for normal moderate-to-vigorous lifestyle activities.

Our results indicate that whilst 150 minutes of activity a week is a useful way to convey the change required at population level because it is inevitably a prescription above ‘baseline’ normal moderate-to-vigorous lifestyle activities, this target will not tally with the picture an individual receives from self-monitoring because this approach inevitably captures these normal moderate-to-vigorous lifestyle activities. Thus, if a patient or healthcare practitioner uses information from self-monitoring and consults widely disseminated physical activity guidance then they will form an erroneous view of their physical activity status (i.e., they are likely to erroneously conclude that their activity is several fold higher than the guidance). Future iterations of physical activity guidelines should either reflect *all* physical activity (i.e., including normal lifestyle activities) or there should be separate guidance for use in conjunction with sophisticated wearable physical activity monitors.

Physical activity guidelines sometimes refer to ‘sustained’ participation in moderate-to-vigorous intensity physical activity undertaken in bouts of 10 minutes or more (Department of Health, 2011). This is often lost in wider dissemination where there is no reference to bouts (e.g., (NHS Choices, 2013)). The concept of a 10-minute period for physical activity accumulation is largely arbitrary (Thompson et al., 2009, Ayabe et al., 2013) and bouts of < 10 minutes are demonstrably very positive for various health outcomes (Glazer et al., 2013, Lagerros and Lagiou, 2007). It is therefore unsurprising that most technology companies have chosen not to build ‘bouts’ into their platforms. Whilst bouts have low relevance to the current analysis because few commercial self-monitoring devices provide this feedback, readers can find an analysis of these data in the supplementary section online (Additional File 3).

Our findings will apply to devices and technologies with a similar accuracy and precision to the instruments that were used in the present study. The multi-sensor devices that we used have excellent validity when compared to doubly-labeled water and/or to criterion measures of energy expenditure in the laboratory (Brage et al., 2005, Brage et al., 2004, Thompson et al., 2006, Crouter et al., 2007, Shuger et al., 2011, Welk et al., 2007, Scheers and Philippaerts, 2013, Johannsen et al., 2010, Bond et al., 2014, Lee et al., 2014). The quality of the output from these devices is quite different to simple accelerometry. This is an important consideration given the rate of technological development – particularly amongst the commercial sector. This is a very rapidly evolving field with tens of millions of devices being sold to consumers and huge investment from global businesses. These commercial instruments are becoming more-and-more sophisticated and include integrated heart rate monitors, gyrometers, and heat and galvanic skin sensors in order to improve the accuracy of estimated energy expenditure. It is noteworthy that the multi-sensor instrument from Bodymedia that we used is already classified by the US Food and Drug Administration (FDA) as a Class II medical device. There was a modest difference between estimates of time engaged in at least moderate intensity activity between comparisons 1 and 2 (~ 13% and 16% of an estimated waking week, respectively). To explore this issue, we examined the effect of age on the differences in the estimates between devices, via an age ∗ device interaction term. A 10-year increment in age decreased the difference in the estimates between devices by 72 minutes (95% CI, 5 to 139 minutes). We regard this as a trivial interaction effect in the face of a mean (SD) for moderate-to-vigorous intensity physical activity overall of 1011 (529) minutes. No other study characteristic, including wear time, substantially influenced the difference between estimates. It appears therefore that the small difference in the estimates might be due to device-specific measurement differences. Importantly, the overall conclusions from the current study are strengthened by the finding that two different multi-sensor devices that capture energy expenditure in distinctive ways and are worn in different body locations produced similar overall estimates of moderate-to-vigorous intensity physical activity.

There will inevitably be some specific considerations that could affect any comparison with the current analysis, such as the threshold used for moderate intensity physical activity. We have used an absolute threshold of 3 METs since this includes most forms of walking (Ainsworth et al., 2011) and is ubiquitous amongst most physical activity guidelines (Thompson et al., 2009). Whilst it would be theoretically possible to shift this MET threshold upwards to reduce the amount of reported activity to a level closer to 150 minutes a week, it would be inappropriate to meddle with what is meant by moderate intensity physical activity to try and force a fit with existing recommendations. We should also highlight that these findings will also only apply when the wear time is as high as reported in the present study, in contrast to surveys that often report a minimum wear time for a valid day of only 10 h (Troiano et al., 2008, Craig et al., 2009). Wear time in most research studies is rarely as high as reported here but we believe that this will become more commonplace as devices become more comfortable to wear or are body mounted. A further consideration is that we have focused on one single physical activity outcome (moderate-to-vigorous intensity activity), but other dimensions are also demonstrably important (Thompson et al., 2015). Future recommendations should take into account the other physical activity dimensions to provide a more complete and holistic view of an individual's physical activity since we do not all have to do the same thing to obtain the health benefits of physical activity (Thompson et al., 2015). However, for the key issue of moderate-to-vigorous intensity physical activity, which is likely to feature in future recommendations as well as feedback from self-monitoring, our analysis helps interpret the output from wearable devices.

## 5 Conclusions

The emergence of affordable wearable technologies to enable the self-monitoring of physical activity has created many exciting opportunities. However, without adjustment, feedback from accurate physical activity monitors is not compatible with widely disseminated physical activity guidance (i.e., the recommendation to accumulate 150 minutes per week of moderate-to-vigorous intensity activity). After taking into account normal moderate-to-vigorous lifestyle activities, an appropriate weekly target is approximately 1000 minutes of moderate-to-vigorous intensity (equivalent to ~ 15% of weekly waking time).

The following are the supplementary data related to this article.

Individual-level data for Comparison 1 Additional File 1

Individual-level data for Comparison 2 Additional File 2

Analysis of physical activity in bouts of 10 minutes Additional File 3

## Authors' contributions

DT conceived the study and drafted the manuscript and is the guarantor. AMB participated in the design of the study and undertook data analysis. OJP participated in the design of the study and collected/collated data. MW collected physical activity data. RB participated in the design of the study and collected clinical data. All authors contributed to the drafting of the manuscript and approved the final version.

## Conflicts of interest

The authors declare that there are no conflicts of interest.

## Transparency document

Transparency document. Image 1

## Figures and Tables

**Table 1:** Participant characteristics.

	Comparison 1 Research instrument (n = 101)	Comparison 2 Commercial instrument (n = 204)
Age, y	51 (6)	64 (6)
Male sex N (% sample)	101 (100)	134 (66)
Height, m	1.79 (0.06)	1.71 (0.09)
Weight, kg	88 (12)	84 (15)
BMI, kg/m^2^	27.5 (3.1)	28.7 (4.5)
Waist circumference, cm	95.7 (10.4)	99.3 (11.1)
Systolic blood pressure, mm Hg	132 (15)	132 (17)
Diastolic blood pressure, mm Hg	87 (11)	87 (11)
TC/HDL cholesterol	4.20 (0.92)	3.92 (1.11)
QRISK, %	5.3 (2.7)	14.2 (6.4)
QDiabetes, %	5.5 (3.2)	12.7 (9.1)
Low Risk, N (% sample)	81 (80)	13 (6)
Moderate risk, N (% sample)	20 (20)	129 (63)
High risk, N (% sample)	0 (0)	62 (30)
PAL, TEE/RMR	1.74 (0.20)	1.77 (0.25)
< 3 METs, minutes/wk	9228 (386)	8990 (571)
> 3 METs, minutes/wk	852 (386)	1090 (571)
> 6 METs, minutes/wk	65 (83)	109 (148)
On body time, %	96 (3)	99 (1)

TC: total cholesterol, PAL: Physical Activity Level, TEE: Total Energy Expenditure, RMR: resting metabolic rate.
