# Supplementary material for: Feedback from physical activity monitors is not compatible with current recommendations: A recalibration study
Source: Prev Med. 2016 Oct;91:389–94. doi: 10.1016/j.ypmed.2016.06.017 (PMC5061550; doi:10.1016/j.ypmed.2016.06.017)
Supplement: Individual-level data for Comparison 1 [file mmc1.pdf]

COMPARISON 1														Physical activity			Activity bouts	
Count	Sex	Age	Height	Weight	BMI	QRisk	QDiabetes	RMR	TEE	AEE	DIT	PAL	On-body time	<3 METs	>3 METs	>6 METs	>3 METs	>6 METs
	M/F	y	m	kg	kg/m^2	%	%	kcal/day	kcal/day	kcal/day	kcal/day	ratio	%	mins/week	mins/week	mins/week	mins/week	mins/week
1	M	48	1.80	88.0	27.3	3.7	4.0	1870	3445	1231	344	1.84	92	9144	936	9	275	0
2	M	54	1.93	104.7	28.0	5.7	6.6	2070	3645	1210	365	1.76	90	9008	1072	7	269	0
3	M	62	1.78	95.1	29.9	11.2	11.1	1722	2774	775	277	1.61	91	9643	437	24	76	10
4	M	55	1.81	101.2	30.9	7.4	10.6	2033	3275	914	328	1.61	95	9644	436	0	142	0
5	M	55	1.83	78.6	23.4	5.9	2.7	1789	2921	839	292	1.63	94	9294	786	63	202	0
6	M	57	1.87	96.7	27.7	7.4	6.8	1670	2677	739	268	1.60	98	9527	553	37	132	0
7	M	57	1.76	76.3	24.7	7.6	3.6	1755	3199	1124	320	1.82	90	9128	952	10	433	0
8	M	47	1.83	97.6	29.3	4.2	5.6	2007	4097	1680	410	2.04	91	8650	1430	20	240	0
9	M	45	1.77	91.8	29.2	3.0	5.0	1925	3657	1366	366	1.90	91	8995	1085	45	355	44
10	M	54	1.78	85.1	27.0	5.2	5.1	1858	3652	1428	365	1.97	91	8961	1119	10	468	0
11	M	51	1.77	90.5	28.8	4.7	6.3	1938	3622	1321	362	1.87	96	9113	967	43	333	0
12	M	54	1.81	87.8	26.9	6.1	5.1	1903	3464	1215	346	1.82	94	9135	945	13	135	0
13	M	46	1.81	107.1	32.7	3.3	9.3	2132	4283	1723	428	2.01	95	8855	1225	37	285	14
14	M	56	1.79	92.8	29.1	6.2	8.2	1934	2905	680	291	1.50	92	9742	338	18	90	15
15	M	60	1.74	78.2	25.8	10.2	5.1	1786	2669	616	267	1.49	96	9234	846	11	168	0
16	M	54	1.87	100.5	28.8	5.5	7.4	2037	3993	1557	399	1.96	97	8670	1410	7	401	0
17	M	58	1.80	89.5	27.6	8.0	7.0	1909	3297	1058	330	1.73	98	9650	430	6	78	0
18	M	53	1.80	87.4	27.0	4.2	4.9	1871	2845	1171	284	1.52	94	9531	549	18	140	0
19	M	50	1.90	85.5	23.8	3.8	2.1	1881	2755	599	276	1.46	92	9741	339	21	49	0
20	M	47	1.76	86.0	27.6	4.2	4.2	1847	2780	655	278	1.51	91	9835	245	7	0	0
21	M	58	1.78	94.3	29.9	7.6	9.7	1953	3318	1033	332	1.70	92	9239	841	33	200	0
22	M	62	1.83	89.6	26.8	11.1	6.6	1643	2661	695	266	1.62	91	9409	671	5	124	0
23	M	52	1.75	91.1	29.7	7.8	7.9	1894	3500	1256	350	1.85	98	9211	869	321	408	260
24	M	52	1.86	112.1	32.4	5.2	11.8	2157	3508	1000	351	1.63	95	9512	568	34	153	21
25	M	59	1.73	88.2	29.5	7.9	9.5	1880	3042	858	304	1.62	97	9342	738	49	346	10
26	M	53	1.75	83.9	27.3	6.1	5.5	1834	3249	1090	325	1.77	97	9114	966	47	282	0
27	M	51	1.80	108.4	33.5	6.4	12.9	2107	4094	1578	409	1.94	100	8745	1335	8	380	0
28	M	53	1.70	90.6	31.3	5.6	10.9	1923	3436	1169	344	1.79	97	9295	785	10	277	0
29	M	46	1.79	91.8	28.7	3.7	4.7	1913	3871	1571	387	2.02	95	8875	1205	3	431	0
30	M	61	1.77	71.8	22.9	10.0	3.0	1453	2530	824	253	1.74	93	9094	986	23	387	0
31	M	52	1.69	68.2	24.0	5.3	2.5	1666	2867	914	287	1.72	93	9381	699	3	130	0
32	M	49	1.77	96.6	30.8	4.8	8.4	1978	3879	1513	388	1.96	97	8638	1442	4	474	0
33	M	51	1.75	86.5	28.4	4.7	6.0	1856	3629	1410	363	1.96	94	9202	878	28	221	0
34	M	48	1.79	102.2	31.9	3.5	9.1	2037	3128	778	313	1.54	97	9594	486	10	35	0
35	M	55	1.90	88.7	24.6	5.6	3.4	1906	3403	1157	340	1.79	94	9271	809	32	239	0
36	M	61	1.73	82.4	27.5	10.8	7.1	1551	2829	994	283	1.82	93	9085	995	49	336	11
37	M	54	1.80	83.7	25.8	6.2	4.3	1834	3188	1035	319	1.74	96	9564	516	9	110	0
38	M	50	1.84	96.6	28.5	4.2	5.9	1965	3779	1436	378	1.92	92	8754	1326	71	513	50
39	M	46	1.95	129.9	34.2	3.8	11.4	2361	3823	1078	382	1.62	92	9299	781	27	274	0
40	M	47	1.64	88.6	32.9	4.2	10.4	1898	3505	1256	350	1.85	91	9097	983	15	310	0
41	M	61	1.76	90.8	29.3	8.6	10.0	1649	2264	635	226	1.37	98	9552	528	13	62	0
42	M	50	1.96	103.0	26.8	4.1	4.2	2050	3574	1167	357	1.74	97	9265	815	28	140	0
43	M	56	1.86	93.0	27.0	8.2	5.6	1956	3445	1145	345	1.76	96	9287	793	94	345	86
44	M	50	1.87	95.3	27.3	4.1	4.5	1985	3684	1330	368	1.86	99	9095	985	7	341	0

COMPARISON 1														Physical activity			Activity bouts	
Count	Sex	Age	Height	Weight	BMI	QRisk	QDiabetes	RMR	TEE	AEE	DIT	PAL	On-body time	<3 METs	>3 METs	>6 METs	>3 METs	>6 METs
	M/F	y	m	kg	kg/m^2	%	%	kcal/day	kcal/day	kcal/day	kcal/day	ratio	%	mins/week	mins/week	mins/week	mins/week	mins/week
45	M	45	1.82	97.3	29.4	2.7	4.9	1994	3126	820	313	1.57	95	9431	649	7	184	0
46	M	52	1.88	102.4	29.0	4.6	6.8	2052	3179	809	318	1.55	97	9332	748	14	206	0
47	M	50	1.77	71.7	23.0	4.3	1.8	1696	2696	731	270	1.59	98	9503	577	22	72	0
48	M	53	1.78	85.5	27.0	5.6	4.9	1847	3396	1211	340	1.84	97	9018	1062	6	243	0
49	M	57	1.76	72.0	23.2	10.0	2.8	1698	2608	650	261	1.54	96	9563	517	4	281	0
50	M	56	1.75	71.7	23.3	8.2	2.8	1698	3438	1396	344	2.02	98	8851	1229	10	344	0
51	M	55	1.75	77.7	25.5	7.1	4.0	1766	2891	836	289	1.64	99	9395	685	17	288	0
52	M	47	1.77	96.8	31.0	3.1	7.5	1984	3608	1263	361	1.82	96	9115	965	18	220	0
53	M	57	1.76	95.0	30.5	8.0	11.0	1961	3604	1283	360	1.84	98	8972	1108	19	342	0
54	M	55	1.74	89.6	29.6	8.4	8.9	1899	3078	871	308	1.62	97	9557	523	41	124	33
55	M	46	1.67	76.0	27.3	2.5	3.6	1743	3866	2122	387	2.22	98	8382	1698	131	835	36
56	M	45	1.89	85.0	23.8	2.0	1.6	1858	3169	1309	317	1.71	98	9145	935	192	405	33
57	M	57	1.83	77.0	23.0	5.4	2.6	1755	3002	1247	300	1.71	95	9495	585	156	395	150
58	M	57	1.77	80.0	25.5	6.6	4.5	1789	3437	1647	344	1.92	99	9221	859	231	353	187
59	M	61	1.72	63.0	21.3	7.7	2.2	1594	2647	1052	265	1.66	96	9301	779	237	404	166
60	M	47	1.82	83.0	25.1	2.3	2.4	1824	3151	1327	315	1.73	98	9310	770	214	410	161
61	M	46	1.78	78.0	24.6	1.9	2.0	1652	2931	1280	293	1.77	97	9362	718	241	323	233
62	M	49	1.79	77.5	24.2	3.7	2.3	1766	3027	3027	303	1.71	100	9507	573	245	338	239
63	M	59	1.82	89.0	26.9	9.4	6.1	1892	3023	1130	302	1.60	98	9180	900	123	277	37
64	M	56	1.79	86.0	26.8	8.1	5.5	1858	2857	999	286	1.54	96	9541	539	8	112	0
65	M	61	1.76	80.0	25.8	12.2	5.4	1523	2242	718	224	1.47	96	9721	359	2	0	0
66	M	48	1.74	104.0	34.4	5.1	12.8	2122	4038	1916	404	1.90	96	8977	1103	64	131	0
67	M	54	1.78	88.0	27.8	7.4	6.1	1973	3150	1177	315	1.60	97	9545	535	4	72	0
68	M	55	1.83	69.0	20.6	4.7	1.4	1663	3336	1673	334	2.01	99	8757	1323	86	449	19
69	M	56	1.81	87.0	26.6	7.8	5.2	1870	2973	1104	297	1.59	95	9654	426	6	92	0
70	M	59	1.80	80.0	24.7	9.4	4.0	1789	2731	941	273	1.53	97	9606	474	17	116	0
71	M	49	1.86	94.0	27.2	3.9	4.2	1950	3534	1231	353	1.81	96	9183	897	13	232	0
72	M	46	1.85	83.0	24.3	3.3	1.9	1824	2891	778	289	1.59	99	9322	758	28	636	0
73	M	54	1.80	85.0	26.2	6.5	4.5	1812	3179	1049	318	1.75	96	9135	945	32	373	10
74	M	56	1.80	101.0	31.2	8.5	11.5	2019	3410	1050	341	1.69	98	9616	464	3	24	0
75	M	59	1.76	83.0	26.8	11.0	6.8	1824	3116	981	297	1.71	97	9507	573	4	115	0
76	M	41	1.78	76.7	24.2	1.2	1.3	1716	2942	931	294	1.71	98	9425	655	130	241	106
77	M	52	1.69	80.2	28.1	5.9	5.8	1793	2459	420	246	1.37	97	9892	188	96	122	73
78	M	49	1.71	75.5	25.8	2.6	3.1	1718	3761	1666	376	2.19	96	8233	1847	176	889	54
79	M	47	1.75	78.9	25.7	2.8	2.8	1630	2889	970	289	1.77	93	9152	928	79	265	0
80	M	51	1.77	86.1	27.4	5.4	5.0	2086	3532	1093	353	1.69	96	9446	634	8	298	0
81	M	36	1.76	89.7	29.1	0.8	2.2	1924	4144	1806	414	2.15	100	8448	1632	280	786	169
82	M	48	1.90	109.1	30.2	3.5	7.0	1661	2932	978	293	1.77	99	9179	901	117	180	80
83	M	52	1.86	107.9	31.1	4.8	10.0	1845	2744	625	274	1.49	98	9466	614	22	299	0
84	M	49	1.82	99.6	30.2	3.6	7.4	1809	2742	658	274	1.52	96	9604	476	79	190	48
85	M	50	1.65	98.0	35.8	5.8	16.5	1997	3311	983	331	1.66	98	9399	681	126	253	105
86	M	38	1.79	75.0	23.5	0.7	0.8	1793	3367	1237	337	1.88	99	8999	1081	153	456	27
87	M	43	1.72	68.1	22.9	1.4	1.1	1499	3720	1849	372	2.48	98	7600	2480	303	1296	185
88	M	35	1.73	71.7	23.8	0.4	0.7	2082	3441	1015	344	1.65	96	9479	601	84	204	35

COMPARISON 1														Physical activity			Activity bouts	
Count	Sex	Age	Height	Weight	BMI	QRisk	QDiabetes	RMR	TEE	AEE	DIT	PAL	On-body time	<3 METs	>3 METs	>6 METs	>3 METs	>6 METs
	M/F	y	m	kg	kg/m^2	%	%	kcal/day	kcal/day	kcal/day	kcal/day	ratio	%	mins/week	mins/week	mins/week	mins/week	mins/week
89	M	50	1.86	85.0	24.7	5.0	2.6	1692	2845	869	285	1.68	95	9388	692	121	419	119
90	M	48	1.78	81.9	25.8	3.3	3.0	1654	2984	1032	298	1.80	97	9051	1029	118	494	54
91	M	47	1.87	76.0	21.9	2.1	1.2	1496	2571	818	257	1.72	95	9263	817	84	266	56
92	M	47	1.76	78.6	25.5	3.2	2.8	1554	2590	777	259	1.67	97	9332	748	155	340	89
93	M	54	1.72	73.6	24.9	4.3	3.5	1496	2513	766	251	1.68	98	9358	722	84	158	76
94	M	45	1.80	89.9	27.7	2.7	3.7	1606	2703	827	270	1.68	96	9015	1065	0	248	0
95	M	51	1.76	81.5	26.4	4.0	4.1	1573	2629	793	263	1.67	93	9337	743	274	384	168
96	M	35	1.78	111.6	35.2	1.2	6.2	2282	3800	1138	380	1.67	96	9282	798	41	103	14
97	M	41	1.84	97.5	28.9	1.7	3.2	2039	3517	1126	352	1.72	97	9211	869	62	331	23
98	M	44	1.95	114.0	29.9	2.8	5.3	2108	2902	503	290	1.38	93	9830	250	1	38	0
99	M	55	1.90	92.0	25.5	5.5	4.1	1704	2836	848	284	1.66	95	9414	666	73	183	18
100	M	44	1.73	81.5	27.2	4.0	3.0	1781	3203	1102	320	1.80	96	8880	1200	18	323	0
101	M	37	1.73	93.2	31.1	0.9	3.7	1859	4660	2335	466	2.51	99	7766	2314	370	1082	155
	Mean	51	1.79	88.4	27.5	5.3	5.5	1846	3220	1125	322	1.74	96	9228	852	65	287	34
	SD	6	0.06	11.7	3.1	2.7	3.2	176	473	402	47	0.20	3	386	386	83	209	62
	Min	35	1.64	63.0	20.6	0.4	0.7	1453	2242	420	224	1.37	90	7600	188	0	0	0
	Max	62	1.96	129.9	35.8	12.2	16.5	2361	4660	3027	466	2.51	100	9892	2480	370	1296	260
