# Supplementary material for: Feedback from physical activity monitors is not compatible with current recommendations: A recalibration study
Source: Prev Med. 2016 Oct;91:389–94. doi: 10.1016/j.ypmed.2016.06.017 (PMC5061550; doi:10.1016/j.ypmed.2016.06.017)
Supplement: Individual-level data for Comparison 2 [file mmc2.pdf]

COMPARISON 2														Physical activity			Activity bouts	
Count	Sex	Age	Height	Weight	BMI	QRisk	QDiabetes	RMR	TEE	AEE	DIT	PAL	On-body time	<3 METs	>3 METs	>6 METs	>3 METs	>6 METs
	M/F	y	m	kg	kg/m^2	%	%	kcal/day	kcal/day	kcal/day	kcal/day	ratio	%	mins/week	mins/week	mins/week	mins/week	mins/week
1	M	68	1.76	96.4	31.1	18.1	22.6	1751	2790	1039	279	1.59	98	9394	686	56	413	10
2	M	67	1.63	73.2	27.7	17.0	9.3	1409	2838	1428	284	2.01	98	8040	2040	151	1733	55
3	M	43	1.74	108.8	35.9	3.6	10.0	2119	3572	1453	357	1.69	99	9094	986	3	620	0
4	F	61	1.63	99.0	37.3	8.3	15.2	1544	2758	1214	276	1.79	95	8945	1135	17	611	0
5	M	58	1.77	89.6	28.6	14.0	10.1	1899	3046	1147	305	1.60	99	9434	646	9	283	0
6	F	71	1.66	82.5	29.9	14.9	26.4	1428	2337	909	234	1.64	99	9225	855	32	562	0
7	F	51	1.62	88.5	33.7	8.2	24.7	1564	2428	864	243	1.55	100	9329	751	75	501	40
8	F	66	1.71	70.5	24.3	9.2	4.2	1354	2022	668	202	1.49	96	9723	357	4	178	0
9	F	69	1.59	81.5	32.2	11.6	23.6	1388	2237	849	224	1.61	99	9638	442	19	159	0
10	M	59	1.82	96.6	29.2	13.2	16.3	1979	3392	1413	339	1.71	99	9034	1046	142	826	70
11	M	63	1.74	74.0	24.6	12.2	4.6	1524	2838	1314	284	1.86	97	8561	1519	147	1100	21
12	M	66	1.82	87.4	26.4	14.6	6.7	1728	3899	2171	390	2.26	99	7968	2112	404	1614	30
13	M	65	1.71	69.9	23.9	14.6	4.1	1462	3018	1556	302	2.06	99	8292	1788	319	1425	121
14	M	69	1.73	91.2	30.6	19.8	27.8	1670	3151	1481	315	1.89	100	8369	1711	66	1376	0
15	F	58	1.66	102.1	37.1	5.5	15.3	1675	2787	1112	279	1.66	99	9277	803	11	308	0
16	M	68	1.76	78.5	25.3	23.9	6.2	1589	2195	607	220	1.38	99	9756	324	2	176	0
17	F	58	1.65	97.5	36.0	6.1	22.8	1637	2204	567	220	1.35	97	9774	306	1	152	0
18	F	56	1.59	84.0	33.1	3.5	16.2	1528	2465	937	246	1.61	98	9178	902	34	504	0
19	F	67	1.71	70.0	24.1	11.1	8.1	1350	2014	664	201	1.49	100	9660	420	7	231	0
20	F	58	1.66	86.0	31.4	6.6	15.5	1544	2557	1013	256	1.66	96	9333	747	15	363	0
21	F	64	1.70	123.7	43.1	7.5	34.0	1769	2733	964	273	1.54	99	9790	290	0	82	0
22	M	66	1.76	70.5	22.8	10.5	3.0	1516	2511	994	251	1.66	99	9247	833	56	515	11
23	M	71	1.78	82.7	26.1	27.0	13.0	1646	3328	1682	333	2.02	100	8478	1602	220	1194	15
24	M	62	1.72	70.9	24.0	9.7	3.5	1481	2207	726	221	1.49	100	9650	430	74	200	24
25	M	67	1.66	66.3	24.1	16.3	7.6	1381	3227	1846	323	2.34	99	7673	2407	500	1784	163
26	M	64	1.84	84.8	25.2	11.2	4.5	1719	2959	1240	296	1.72	99	9198	882	72	630	0
27	M	67	1.80	89.4	27.6	13.3	14.5	1727	2782	1055	278	1.61	99	9409	671	28	302	0
28	F	67	1.67	67.3	24.3	8.8	3.2	1310	2507	1197	251	1.91	99	8493	1587	105	1140	13
29	M	63	1.77	81.1	26.0	10.2	11.3	1617	2965	1348	297	1.83	99	8792	1288	99	870	0
30	F	62	1.67	74.0	26.5	7.9	7.5	1366	2168	802	217	1.59	98	9429	651	34	425	0
31	M	68	1.68	69.4	24.7	14.3	5.6	1424	2785	1362	279	1.96	97	8381	1699	175	1304	22
32	M	69	1.69	75.4	26.6	24.8	10.1	1488	3268	1781	327	2.20	99	8203	1877	263	1237	99
33	F	70	1.63	93.0	35.0	14.4	23.9	1497	2233	736	223	1.49	100	9702	378	19	124	0
34	M	60	1.98	112.8	28.8	14.0	7.5	2165	3829	1664	383	1.77	100	9038	1042	18	562	0
35	M	52	1.93	101.7	27.3	17.4	4.6	2037	3404	1367	340	1.67	100	9251	829	39	546	19
36	M	64	1.73	61.6	20.6	15.9	6.2	1406	2141	735	214	1.52	99	9592	488	124	316	60
37	M	69	1.78	82.0	25.9	16.6	6.5	1640	3336	1696	334	2.03	100	8513	1567	199	1255	99
38	M	62	1.76	107.3	34.8	12.4	18.2	1845	3110	1264	311	1.69	100	9054	1026	37	671	0
39	M	57	1.73	92.5	30.9	6.7	22.6	1932	3690	1758	369	1.91	98	8438	1642	60	1138	30
40	M	64	1.79	71.5	22.3	10.7	4.2	1554	3825	2271	383	2.46	98	7622	2458	808	1884	325
41	F	71	1.58	64.4	26.0	9.3	3.9	1246	3053	1807	305	2.45	99	7578	2502	638	1928	0
42	M	62	1.73	88.1	29.4	11.8	9.2	1647	3144	1497	314	1.91	99	8562	1518	21	945	0
43	F	71	1.57	73.8	30.1	14.0	18.5	1317	2059	743	206	1.56	100	9466	614	30	328	0
44	M	66	1.71	113.0	38.6	16.0	41.6	1853	3417	1564	342	1.84	99	8506	1574	2	802	0

**COMPARISON 2**														Physical activity			Activity bouts	
Count	Sex	Age	Height	Weight	BMI	QRisk	QDiabetes	RMR	TEE	AEE	DIT	PAL	On-body time	<3 METs	>3 METs	>6 METs	>3 METs	>6 METs
	M/F	y	m	kg	kg/m^2	%	%	kcal/day	kcal/day	kcal/day	kcal/day	ratio	%	mins/week	mins/week	mins/week	mins/week	mins/week
45	M	61	1.72	81.9	27.7	15.4	12.3	1581	2341	761	234	1.48	99	9466	614	87	413	0
46	M	70	1.78	92.2	29.1	18.8	17.5	1733	3504	1771	350	2.02	100	8292	1788	269	1556	120
47	F	66	1.66	83.0	30.1	8.7	10.2	1432	2524	1092	252	1.76	99	9232	848	6	397	0
48	F	64	1.56	80.4	33.0	10.4	16.3	1366	2233	867	223	1.63	99	9408	672	22	364	0
49	F	70	1.62	56.3	21.4	10.3	2.2	1205	2228	1023	223	1.85	100	9084	996	405	644	234
50	M	44	1.85	119.4	34.9	10.3	12.8	2241	5014	2773	501	2.24	99	7488	2592	28	2041	23
51	M	65	1.78	86.0	27.3	13.5	8.3	1671	2813	1141	281	1.68	98	9159	921	66	538	0
52	M	67	1.68	68.0	24.1	15.8	4.5	1416	3236	1821	324	2.29	99	8037	2043	575	1570	312
53	M	68	1.76	89.6	28.9	15.1	20.1	1689	3246	1556	325	1.92	99	8475	1605	21	1229	0
54	M	70	1.82	74.8	22.7	13.5	6.7	1609	3004	1395	300	1.87	99	9054	1026	119	715	10
55	M	59	1.74	65.6	21.6	7.2	2.5	1624	3643	2019	364	2.24	100	8261	1819	573	1455	226
56	M	64	1.81	89.0	27.3	24.8	17.2	1730	3105	1375	310	1.79	98	9053	1027	42	640	26
57	M	70	1.69	80.1	28.2	25.7	9.6	1530	2690	1160	269	1.76	97	8997	1083	132	737	26
58	M	57	1.80	95.5	29.5	17.2	13.1	1967	3479	1512	348	1.77	98	9062	1018	14	521	0
59	M	61	1.82	99.3	30.0	14.9	11.1	1836	4130	2295	413	2.25	100	8136	1944	82	1120	0
60	M	54	1.73	94.1	31.4	14.3	24.5	1951	3151	1200	315	1.62	98	9370	710	44	413	0
61	M	69	1.78	91.3	28.8	16.9	10.1	1724	3735	2011	374	2.17	98	8012	2068	194	1465	50
62	M	67	1.81	102.7	31.3	29.6	20.6	1857	2879	1022	288	1.55	99	9388	692	19	361	0
63	F	70	1.58	84.6	33.9	14.2	14.8	1408	2306	898	231	1.64	99	9606	474	28	237	0
64	F	68	1.65	76.5	28.1	13.2	11.3	1376	2153	777	215	1.56	99	9534	546	35	303	0
65	M	69	1.74	86.7	28.6	17.8	9.5	1644	2801	1157	280	1.70	100	9015	1065	115	844	0
66	M	63	1.84	84.3	24.8	11.9	3.9	1723	2831	1108	283	1.64	100	9235	845	10	699	0
67	F	69	1.61	54.6	21.0	12.4	2.6	1186	2382	1197	238	2.01	98	8520	1560	419	1198	217
68	F	70	1.64	89.3	33.4	12.3	18.6	1470	2741	1271	274	1.86	99	9161	919	10	372	0
69	F	60	1.68	60.9	21.5	8.2	1.8	1269	2346	1077	235	1.85	99	9136	944	220	510	60
70	M	70	1.73	81.3	27.2	20.5	10.8	1585	2739	1154	274	1.73	96	9271	809	22	411	0
71	M	66	1.77	74.1	23.8	11.9	4.2	1554	2781	1227	278	1.79	99	8976	1104	82	798	30
72	M	64	1.82	91.0	27.5	13.4	7.8	1760	2848	1088	285	1.62	98	9056	1024	42	633	0
73	M	64	1.78	97.2	30.7	9.7	10.9	1778	3259	1481	326	1.83	99	8719	1361	10	836	0
74	M	59	1.75	85.7	28.1	12.5	13.1	1854	4010	2155	401	2.16	99	8170	1910	285	1303	151
75	M	60	1.71	79.6	27.2	9.0	6.5	1492	3424	1932	342	2.30	99	7533	2547	170	1706	63
76	M	58	1.75	103.0	33.6	14.8	16.8	2053	3929	1876	393	1.91	99	8450	1630	87	1156	78
77	M	66	1.69	87.5	30.8	21.0	14.9	1598	2411	813	241	1.51	100	9559	521	12	245	0
78	M	63	1.80	85.5	26.4	11.8	6.3	1691	2588	897	259	1.53	97	9505	575	23	408	0
79	M	65	1.65	75.6	27.8	20.5	10.1	1455	3115	1660	312	2.14	98	7860	2220	242	1763	32
80	M	65	1.83	83.5	25.1	10.2	5.3	1697	2546	848	255	1.50	99	9826	254	70	92	69
81	F	71	1.69	76.4	26.8	12.4	5.4	1502	2726	1224	273	1.82	99	8649	1431	67	1016	0
82	M	52	1.72	93.8	31.7	24.3	15.6	1947	4058	2111	406	2.08	99	7937	2143	71	1712	0
83	M	66	1.70	71.5	24.7	16.3	11.2	1467	2966	1499	297	2.02	98	8351	1729	208	1267	45
84	M	65	1.63	65.2	24.5	29.6	7.5	1342	2960	1618	296	2.21	99	7990	2090	618	1818	329
85	M	62	1.70	73.2	25.5	7.8	4.8	1482	3102	1620	310	2.09	99	8405	1675	585	1315	542
86	M	69	1.82	96.6	29.2	20.9	10.9	1811	3064	1252	306	1.69	97	9138	942	7	501	0
87	F	71	1.57	61.0	24.9	15.3	7.0	1217	2317	1099	232	1.90	98	8990	1090	302	592	82
88	M	67	1.82	86.2	25.9	23.8	10.1	1717	3879	2163	388	2.26	99	7867	2213	308	1788	42

COMPARISON 2														Physical activity			Activity bouts	
Count	Sex	Age	Height	Weight	BMI	QRisk	QDiabetes	RMR	TEE	AEE	DIT	PAL	On-body time	<3 METs	>3 METs	>6 METs	>3 METs	>6 METs
	M/F	y	m	kg	kg/m^2	%	%	kcal/day	kcal/day	kcal/day	kcal/day	ratio	%	mins/week	mins/week	mins/week	mins/week	mins/week
89	F	65	1.61	72.1	28.0	7.0	5.7	1323	2196	873	220	1.66	98	9403	677	17	376	0
90	M	62	1.56	87.8	36.1	33.2	76.1	1479	2864	1385	286	1.94	100	8810	1270	157	991	0
91	F	69	1.58	53.8	21.5	11.3	7.5	1165	1827	661	183	1.57	99	9291	789	47	428	11
92	M	68	1.75	81.3	26.5	23.7	7.6	1604	2785	1181	279	1.74	98	9211	869	15	460	0
93	M	65	1.76	106.5	34.6	11.6	19.2	1843	3655	1813	366	1.98	99	8197	1883	63	1310	16
94	M	69	1.79	92.8	29.0	29.5	31.2	1748	3066	1318	307	1.75	99	8867	1213	7	818	0
95	F	66	1.52	63.7	27.8	9.2	9.6	1216	2449	1233	245	2.01	100	8493	1587	329	1032	143
96	F	52	1.63	110.9	41.7	3.8	20.9	1746	2642	896	264	1.51	98	9458	622	1	193	0
97	F	62	1.61	69.8	26.9			1305	2826	1521	283	2.17	99	8378	1702	618	1366	448
98	M	68	1.65	92.8	34.1	19.2	24.9	1612	2179	567	218	1.35	99	9710	370	3	144	0
99	M	68	1.65	55.9	20.5	24.0	6.1	1277	2100	824	210	1.65	96	9174	906	128	494	52
100	M	68	1.85	96.9	28.3	13.9	7.7	1843	2813	969	281	1.53	100	9491	589	29	423	0
101	M	68	1.86	108.2	31.4	20.7	15.7	1955	3210	1255	321	1.64	98	9581	499	0	165	0
102	F	69	1.63	66.3	25.0	10.6	12.1	1287	2024	737	202	1.57	99	9524	556	104	307	52
103	M	70	1.80	107.7	33.2	24.4	28.9	1893	3509	1617	351	1.85	99	8758	1322	57	923	0
104	F	71	1.53	53.5	22.9	10.6	6.2	1140	1678	538	168	1.47	98	9826	254	44	145	20
105	F	60	1.65	92.3	34.1	4.1	21.1	1595	2818	1223	282	1.77	99	8970	1110	19	625	0
106	M	65	1.74	77.9	25.7	11.8	6.2	1564	3674	2110	367	2.35	99	7631	2449	497	1943	151
107	F	71	1.64	66.4	24.8	11.9	4.3	1292	2658	1367	266	2.06	99	8440	1640	416	1159	190
108	M	69	1.77	74.4	23.9	15.6	7.8	1561	2696	1135	270	1.73	99	8942	1138	38	632	0
109	M	62	1.74	78.4	26.0	11.4	6.4	1568	3049	1480	305	1.94	99	8721	1359	163	960	27
110	M	58	1.80	102.0	31.7	8.0	11.7	2041	5023	2982	502	2.46	100	7140	2940	30	2390	0
111	F	56	1.64	80.2	30.0	11.9	16.9	1497	2325	828	232	1.55	99	9386	694	15	323	0
112	F	67	1.68	91.1	32.5	16.1	17.5	1505	2647	1142	265	1.76	99	9322	758	36	400	21
113	F	69	1.68	95.8	34.1	12.6	13.1	1542	2713	1172	271	1.76	100	9081	999	1	442	0
114	F	51	1.78	125.5	39.8	2.5	11.5	1865	3173	1308	317	1.70	99	9414	666	0	211	0
115	M	67	1.77	84.0	27.0	11.9	15.0	1648	2901	1253	290	1.76	98	8876	1204	57	791	0
116	M	70	1.76	84.9	27.4	32.6	13.9	1647	3118	1471	312	1.89	99	8713	1367	128	905	14
117	F	68	1.56	74.1	30.4	9.8	8.5	1316	2580	1264	258	1.96	100	8460	1621	169	948	0
118	M	64	1.78	86.2	27.2	11.7	7.0	1678	2978	1299	298	1.77	99	8922	1158	36	751	0
119	M	71	1.75	85.8	28.0	19.9	10.4	1645	2639	993	264	1.60	97	9493	587	21	212	0
120	F	67	1.66	54.1	19.6	20.5	1.9	1204	2036	831	204	1.69	100	9187	893	154	506	82
121	F	70	1.75	89.3	29.2	13.8	6.4	1523	2440	917	244	1.60	98	9439	641	7	290	0
122	M	57	1.87	108.6	31.1	9.5	10.7	2117	3380	1263	338	1.60	98	9479	601	0	257	0
123	M	66	1.82	96.6	29.2	32.6	13.0	1811	3059	1248	306	1.69	98	9280	800	50	548	18
124	M	70	1.72	90.4	30.6	31.6	19.6	1658	2846	1188	285	1.72	99	9161	919	46	520	0
125	F	61	1.54	82.2	34.6	8.2	27.7	1371	2009	639	201	1.47	98	9514	566	0	257	0
126	M	56	1.87	120.0	34.5	8.1	17.1	2247	4048	1800	405	1.80	99	8835	1245	0	786	0
127	M	62	1.79	94.2	29.6	22.0	10.9	1760	3173	1413	317	1.80	99	8727	1353	52	913	0
128	M	63	1.65	76.2	28.0	11.5	7.0	1461	2283	822	228	1.56	96	9278	802	49	578	0
129	M	66	1.83	91.8	27.4	15.4	11.7	1777	3073	1295	307	1.73	99	9183	897	8	500	0
130	F	65	1.56	79.6	32.7	10.2	10.9	1359	2312	953	231	1.70	99	9114	966	59	562	0
131	M	57	1.81	82.9	25.3	11.4	5.4	1822	3541	1719	354	1.94	99	8658	1422	176	752	0
132	F	67	1.54	84.0	35.7	11.3	17.3	1385	2108	723	211	1.52	99	9770	310	5	78	0

COMPARISON 2														Physical activity			Activity bouts	
Count	Sex	Age	Height	Weight	BMI	QRisk	QDiabetes	RMR	TEE	AEE	DIT	PAL	On-body time	<3 METs	>3 METs	>6 METs	>3 METs	>6 METs
	M/F	y	m	kg	kg/m^2	%	%	kcal/day	kcal/day	kcal/day	kcal/day	ratio	%	mins/week	mins/week	mins/week	mins/week	mins/week
133	M	68	1.62	81.4	31.2	25.0	14.8	1479	2387	908	239	1.61	99	9329	751	60	398	11
134	M	66	1.69	74.8	26.2	14.1	18.4	1487	2976	1489	298	2.00	100	8278	1802	190	1254	42
135	M	54	1.79	95.0	29.6	11.6	14.4	1961	3182	1221	318	1.62	99	9466	614	51	389	21
136	M	49	1.81	123.3	37.8	11.6	32.5	2285	3737	1452	374	1.64	100	9131	949	3	743	0
137	F	70	1.75	81.6	26.8	17.2	10.7	1462	2353	891	235	1.61	99	9300	780	66	535	0
138	M	48	1.81	85.4	26.1	9.5	4.2	1851	4420	2569	442	2.39	98	7212	2868	313	2563	58
139	F	66	1.65	57.0	20.9	10.4	2.3	1222	2045	823	205	1.67	99	9368	712	111	368	27
140	F	59	1.57	83.4	33.8	12.6	27.3	1523	2443	920	244	1.60	99	9273	807	86	458	22
141	F	70	1.49	58.6	26.4	14.3	5.0	1162	1876	715	188	1.62	99	9153	927	203	635	56
142	F	66	1.68	86.6	30.9	14.1	25.3	1469	2179	710	218	1.48	98	9538	542	15	366	0
143	F	58	1.53	76.9	33.1	4.1	18.6	1470	2150	680	215	1.46	99	9595	485	6	168	0
144	M	70	1.83	83.0	24.9	14.4	5.1	1698	2659	961	266	1.57	99	9526	554	117	310	70
145	M	68	1.66	75.2	27.3	18.6	11.4	1462	2210	748	221	1.51	99	9569	511	119	346	84
146	M	61	1.67	79.2	28.6	19.6	15.6	1508	2792	1284	279	1.85	98	8843	1237	51	730	13
147	M	69	1.90	117.8	32.8	19.3	21.1	2081	3575	1494	358	1.72	100	9276	804	3	425	0
148	M	68	1.73	85.8	28.7	18.6	12.9	1626	2384	758	238	1.47	98	9675	405	22	167	0
149	M	68	1.78	84.5	26.7	23.0	6.6	1662	3198	1536	320	1.92	99	8680	1400	208	951	24
150	F	69	1.53	84.0	35.7	16.1	44.7	1380	2125	745	213	1.54	99	9430	650	3	321	0
151	M	65	1.73	97.2	32.5	14.8	17.6	1729	3198	1469	320	1.85	98	8662	1418	76	1030	0
152	M	68	1.86	98.4	28.4	28.6	31.8	1867	3798	1931	380	2.03	99	8307	1773	101	1260	37
153	M	67	1.77	69.8	22.3	15.0	4.7	1519	2772	1253	277	1.82	99	8900	1180	232	810	55
154	F	67	1.55	67.4	28.2	13.2	6.8	1259	2853	1595	285	2.27	98	7992	2088	598	1514	187
155	F	43	1.67	107.7	38.8	3.4	20.2	1720	2549	829	255	1.48	99	9577	503	9	244	0
156	F	61	1.50	52.6	23.5	10.8	3.0	1119	1549	430	155	1.38	99	9782	298	21	101	0
157	M	67	1.83	97.3	29.2	17.2	9.5	1827	3389	1562	339	1.85	99	8629	1451	40	1050	0
158	F	70	1.65	62.9	23.2	12.2	2.8	1269	2430	1161	243	1.92	98	8661	1419	211	918	78
159	M	67	1.71	78.1	26.8	33.1	18.8	1537	2608	1071	261	1.70	98	9000	1080	98	879	28
160	F	63	1.64	84.3	31.3	11.4	12.1	1433	1927	494	193	1.34	98	9857	223	4	86	0
161	M	68	1.76	92.9	30.0	21.1	19.9	1719	2798	1079	280	1.63	98	9202	878	5	506	0
162	M	64	1.75	85.3	27.9	18.7	7.3	1641	2424	783	242	1.48	100	9603	477	3	208	0
163	M	61	1.86	92.9	26.8	19.9	6.8	1817	3446	1629	345	1.90	99	8984	1096	114	654	16
164	M	60	1.60	68.6	26.8	13.6	7.3	1343	3419	2076	342	2.55	96	7960	2120	473	1578	130
165	F	68	1.60	68.4	26.7	12.7	4.9	1289	2128	839	213	1.65	100	9304	776	33	478	0
166	F	45	1.73	99.2	33.2	2.3	13.8	1651	2982	1331	298	1.81	99	8611	1469	22	873	0
167	M	59	1.74	72.0	23.8	10.8	3.8	1697	2926	1228	293	1.72	99	9103	977	260	634	99
168	F	69	1.71	71.5	24.5	10.5	3.5	1364	2274	910	227	1.67	99	9442	638	59	280	0
169	F	56	1.58	93.3	37.3	5.1	25.6	1603	2349	746	235	1.47	98	9615	465	8	185	0
170	F	61	1.60	70.7	27.6	8.9	5.5	1308	2224	916	222	1.70	99	9315	765	94	350	14
171	M	64	1.79	92.3	28.7	13.1	19.3	1743	3234	1491	323	1.86	99	8685	1395	42	965	12
172	M	60	1.88	103.0	29.0	9.5	10.0	1928	3682	1755	368	1.91	99	8767	1313	20	759	0
173	M	68	1.80	89.4	27.5	13.5	8.3	1727	2935	1209	294	1.70	98	9224	856	67	496	53
174	M	48	1.77	116.2	37.1	3.6	31.1	2204	3165	961	317	1.44	100	9809	271	11	113	0
175	M	63	1.79	71.3	22.4	9.4	2.7	1553	2973	1421	297	1.92	100	8550	1530	142	1085	48
176	M	63	1.64	73.3	27.4	12.9	14.8	1420	2656	1236	266	1.87	95	8712	1369	193	819	30

COMPARISON 2														Physical activity			Activity bouts	
Count	Sex	Age	Height	Weight	BMI	QRisk	QDiabetes	RMR	TEE	AEE	DIT	PAL	On-body time	<3 METs	>3 METs	>6 METs	>3 METs	>6 METs
	M/F	y	m	kg	kg/m^2	%	%	kcal/day	kcal/day	kcal/day	kcal/day	ratio	%	mins/week	mins/week	mins/week	mins/week	mins/week
177	M	64	1.71	71.6	24.6	16.4	4.5	1478	2592	1115	259	1.75	100	9079	1001	171	626	28
178	M	69	1.74	91.0	29.9	34.1	19.2	1683	2977	1294	298	1.77	98	8983	1097	18	463	11
179	M	59	1.78	75.9	24.0	11.0	3.8	1742	3187	1445	319	1.83	99	8700	1380	74	913	11
180	F	66	1.67	74.5	26.7	14.4	6.1	1370	2103	734	210	1.54	99	9665	415	84	242	0
181	F	49	1.64	97.0	36.2	1.3	17.9	1866	2979	1113	298	1.60	99	9454	626	3	265	0
182	M	67	1.76	100.4	32.5	25.4	27.1	1787	3284	1497	328	1.84	99	8762	1318	3	785	0
183	M	46	1.72	95.7	32.3	8.1	20.2	1969	3010	1041	301	1.53	100	9421	659	1	364	0
184	F	67	1.53	49.2	21.0	13.2	3.8	1106	1692	586	169	1.53	87	9539	541	67	309	0
185	M	61	1.80	111.3	34.4	12.5	22.9	1925	3268	1342	327	1.70	99	8941	1139	2	668	0
186	F	59	1.63	106.2	40.2	6.9	15.7	1708	2326	618	233	1.36	98	9694	386	0	184	0
187	M	53	1.70	73.2	25.3	9.6	8.0	1711	2641	930	264	1.54	99	9552	528	42	242	0
188	M	69	1.83	86.0	25.6	17.2	5.3	1725	2427	702	243	1.41	99	9861	219	3	79	0
189	M	70	1.79	99.0	31.1	18.5	18.7	1804	3510	1706	351	1.95	99	8625	1455	151	1079	10
190	M	69	1.73	74.9	25.1	14.8	5.2	1525	2336	811	234	1.53	99	9490	590	21	350	0
191	M	62	1.72	79.3	26.8	16.4	7.1	1557	2409	852	241	1.55	99	9593	487	53	295	25
192	M	49	1.67	89.6	32.1	6.7	23.2	1899	3420	1520	342	1.80	99	8638	1442	27	909	0
193	F	70	1.58	62.6	25.1	12.5	4.3	1235	2376	1141	238	1.92	99	8747	1333	349	873	69
194	M	62	1.73	89.0	29.6	13.3	16.3	1659	2800	1141	280	1.69	99	9043	1037	61	752	0
195	M	57	1.81	89.8	27.4	14.0	10.3	1901	3124	1222	312	1.64	100	9270	810	78	536	15
196	M	68	1.93	106.1	28.6	17.5	15.1	2000	3384	1384	338	1.69	97	9167	913	17	584	10
197	M	67	1.77	90.3	28.8	22.7	10.0	1706	3163	1458	316	1.85	99	8573	1507	114	996	99
198	M	66	1.85	88.1	25.9	16.7	5.9	1758	2961	1202	296	1.68	99	9321	759	33	352	0
199	M	59	1.68	77.6	27.5	6.9	6.5	1762	2704	943	270	1.54	94	9435	645	67	349	11
200	F	68	1.55	88.0	36.9	12.9	15.6	1421	2100	679	210	1.48	99	9507	573	7	295	0
201	F	68	1.69	66.9	23.4	11.2	5.4	1319	2374	1056	237	1.80	100	8915	1165	168	731	35
202	F	65	1.56	70.3	29.1	10.0	11.2	1284	2082	798	208	1.62	100	9270	810	84	476	0
203	M	64	1.84	68.2	20.3	10.5	1.8	1568	2855	1287	285	1.82	98	8956	1124	167	784	53
204	M	59	1.73	87.0	29.0	10.5	13.4	1870	3295	1425	329	1.76	98	9088	992	152	606	73
	Mean	64	1.71	84.4	28.7	14.2	12.7	1609	2845	1236	284	1.77	99	8990	1090	109	715	34
	SD	6	0.09	15.0	4.5	6.4	9.1	243	587	436	59	0.25	1	571	571	148	489	74
	Min	43	1.49	49.2	19.6	1.3	1.8	1106	1549	430	155	1.34	87	7140	219	0	78	0
	Max	71	1.98	125.5	43.1	34.1	76.1	2285	5023	2982	502	2.55	100	9861	2940	808	2563	542
