# Supplementary material for: Feedback from physical activity monitors is not compatible with current recommendations: A recalibration study
Source: Prev Med. 2016 Oct;91:389–94. doi: 10.1016/j.ypmed.2016.06.017 (PMC5061550; doi:10.1016/j.ypmed.2016.06.017)
Supplement: Analysis of physical activity in bouts of 10 minutes [file mmc3.docx]

**Additional File 3**

**Analysis of physical activity in bouts of 10 minutes**

**Introduction and Methods**

Some physical activity recommendations refer to the requirement to accumulate physical activity in bouts of 10 minutes or more. The 10-minute bout is largely arbitrary and smaller bouts are demonstrably important for health. Furthermore, a focus on bouts also introduces the problem of how to define what is meant by a bout. For example, three consecutive 8-minute blocks of sustained activity with a 2-3 minute gap in between each block might not count towards a bout-based metric but a single 10-minute block would. Of course, the issue of how to factor in any baseline normal lifestyle physical activity still remains even if physical activity is analysed in bouts. However, given the prominence of bouts in some recommendations, we used in-house software to examine the number of minutes engaged in physical activity above 3 METs in bouts of at least 10 minutes.

**Results**

On average, participants undertook 287 ± 209 and 715 ± 489 minutes of moderate-to-vigorous intensity physical activity in bouts of 10 minutes or more in Comparisons 1 and 2, respectively. Based on this analysis, a PAL of 1.75 equates to 292 minutes (Comparison 1) and 683 minutes (Comparison 2) of moderate-to-vigorous intensity physical activity (Figure 1S). A caveat here is that the residuals for Comparison 1, in particular, are not well behaved, suggesting problems with the specification of the simple linear model. Re-specification of the model as a power function of the form Y=aX^b^ improved the fit but made no material difference to the estimate.


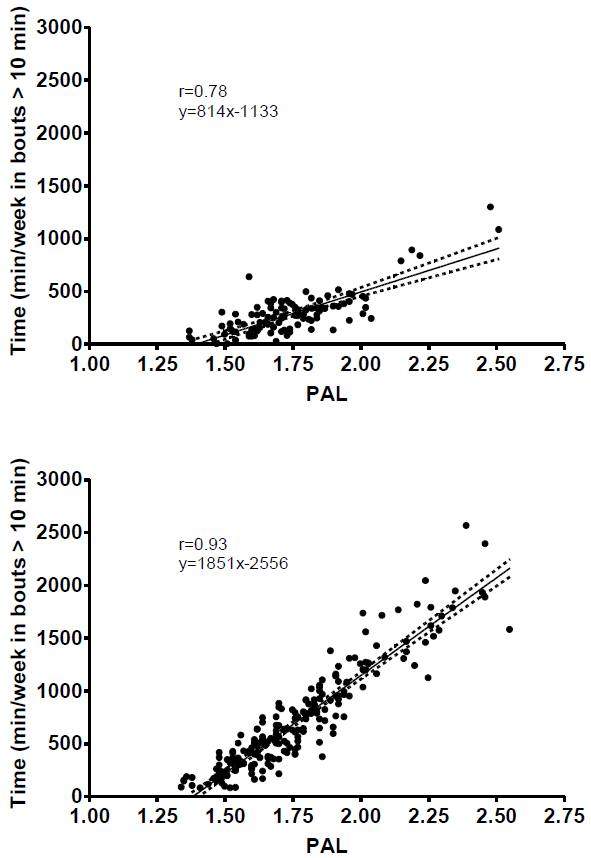


Figure 1S: The relationship between PAL as a standardised measure of physical activity energy expenditure (TEE/BMR) and moderate-to-vigorous intensity physical activity in bouts of 10 minutes or more. Panel A shows the research instrument used in Comparison 1 (n=101) and Panel B the commercial instrument used in Comparison 2 (n=204). The regression line with 95% CI are included.

**Discussion**

As highlighted in the primary discussion, an analysis of physical activity in bouts of 10 minutes is arbitrary and problematic. Nonetheless, it is noteworthy that even in this analysis most participants exceeded weekly physical activity recommendations after taking bouts into consideration. These results are affected by the same issue of how to factor in baseline physical activity (i.e., normal lifestyle activities). According to this analysis, a PAL of 1.75 equates to approximately 2-5 fold the recommendation. It is noteworthy that there was a bigger difference between devices for this bout-related analysis. Although the reason for this is not fully clear, it is likely to be related to the variability (and limitations) of the different types of physiological sensors used in these devices. For example, an unresolved issue with temperature and heat flux sensing (as used in the BodyMedia FIT device) is the lag time to dissipate heat from the body upon completing moderate-to-vigorous intensity activity [1]. This could conceivably ‘extend’ a given bout or mask short gaps between more fragmented activities. As is the case for many commercial instruments, the algorithms used in the BodyMedia device are proprietary and thus it is difficult to determine if this consideration is a main cause of the reported difference. An alternative explanation is that sample-specific differences (e.g., age) could mean that differences in the pattern and nature of activity between comparisons could affect bout-related scores. Whatever the explanation, this does not affect the overall conclusion that an analysis of activity even after factoring in bouts will not be compatible with widely disseminated physical activity recommendations. As stated in the primary discussion, this analysis seems less relevant for the future because so few commercial monitors provide feedback in the form of bouts and many agencies omit bouts in their dissemination of physical activity guidance.

**References**

Chen, K.Y., et al., *Redefining the roles of sensors in objective physical activity monitoring.* Med Sci Sports Exerc, 2012. **44**(1 Suppl 1): p. S13-23.
